# Endoscopic clipping-assisted endoscopic injection sclerotherapy in the treatment of esophageal varices

**DOI:** 10.1055/a-2187-9608

**Published:** 2023-11-14

**Authors:** Bo Wu, Xincheng Xie, Zhuying Zhou, Chaojun Huang, Hai Wang

**Affiliations:** 1 Department of Gastroenterology, Affiliated Hangzhou Xixi Hospital of Zhejiang University School of Medicine, Hangzhou, China; 2 Outpatient Department, Affiliated Hangzhou Xixi Hospital of Zhejiang University School of Medicine, Hangzhou, China

Endoscopic variceal ligation (EVL) and endoscopic injection sclerotherapy (EIS) are important treatments for acute bleeding and secondary prevention of variceal bleeding 1 2 . EVL is known for its lower risk of adverse reactions but is limited to superficial veins and requires a certain diameter of varicose veins 3 . In contrast, EIS can reach deep perforating vessels regardless of vessel diameter. However, EIS has limitations such as rapid dilution of sclerosing agents and an increased risk of distal embolism due to fast blood flow and abundant vasculature. To overcome these challenges, we have combined metal clipping, which has been used for a long time in the treatment of esophageal varices 4 , with EIS to create endoscopic clipping-assisted EIS ( Video 1 ), which improves sclerosing agent retention in varicose veins, reduces the amount used, and decreases the risk of distal embolism.

**Video 1** Animated demonstration and practical procedure of endoscopic clipping-assisted endoscopic injection sclerotherapy. Source for graphical illustrations: Yizheng Fang.


Before a non-emergency endoscopy, patients were advised to fast for 12 hours and underwent anesthesia risk assessment. A transparent cap was attached to the distal end of the gastroscope. The lubricated gastroscope was then inserted into the esophagus, and a target vessel was selected approximately 30 cm from the incisors. Using a metal clip, the vein was ligated, and a puncture needle was inserted near the pectinate line. After withdrawing blood, an injection mixture of 1 % polidocanol (Guoyao Zhunzi H20080445, Tianyu Changʼan Group, Xi’an, China) and methylene blue in a 10:0.1 ratio was injected internally. Another metal clip was used to close the puncture site ( Fig. 1 ). The total dose of polidocanol injection administered in a single treatment did not exceed 40 ml.

**Fig. 1 a:**
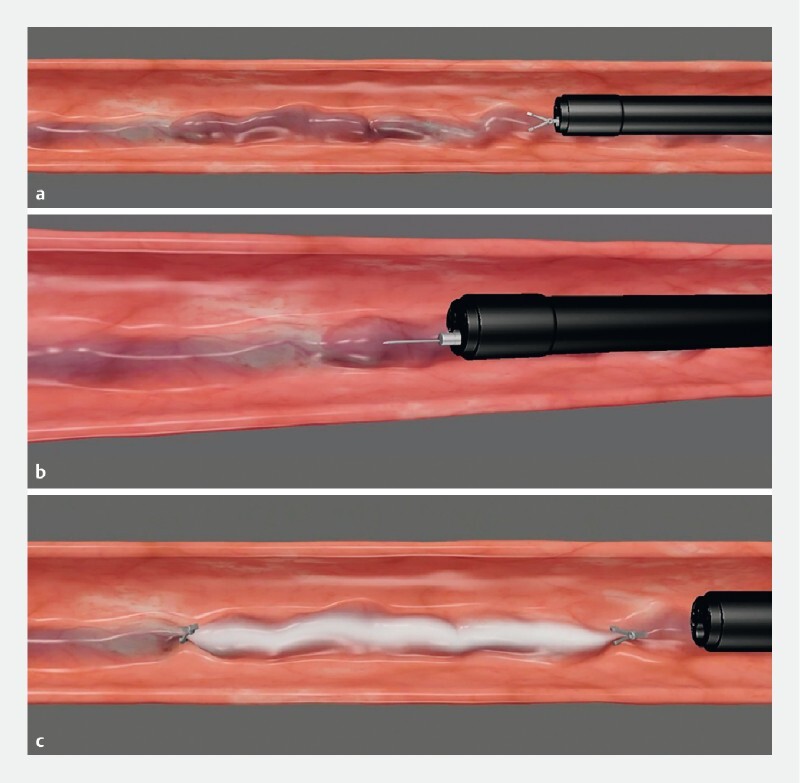
Ligated the proximal end of the varicose vein with a metal clip. **b** Injected sclerosing agent above the pectinate line. **c** Used another metal clip to close the puncture site. Source: Yizheng Fang.

We treated 22 patients using the endoscopic clipping-assisted EIS method and there were no severe complications. One patient with advanced liver cancer discontinued treatment due to primary disease after more than 1 month after surgery, whereas the remaining 21 patients showed significant improvement in varicose veins ( Fig. 2 ). The average amount of sclerosing agent used was 22 ± 8 ml, and the average cost of treatment related to esophageal varices was 2449 ± 741 renminbi (RMB). During the same period, the average amount of sclerosant used in pure EIS treatment at our center was 31 ± 13 ml, with an average treatment cost of 6080 ± 4329 RMB.

**Fig. 2 a:**
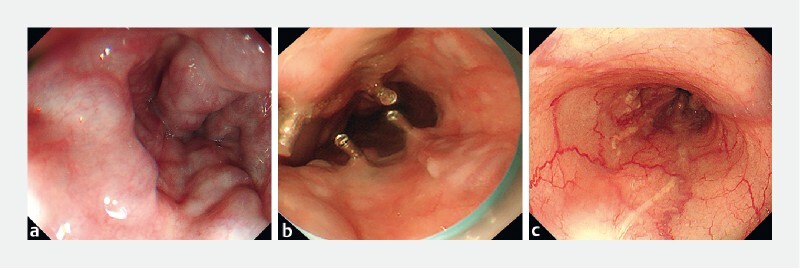
Gastroscopy revealed severe esophageal varices. **b** Endoscopic clipping-assisted endoscopic injection sclerotherapy was used to treat varicose veins. **c** The postoperative re-examination indicated that the varicose veins were basically eliminated.

Endoscopy_UCTN_Code_TTT_1AO_2AD

## Notes

Endoscopy E-Videos

https://eref.thieme.de/e-videos
*E-Videos* is an open access online section of the journal *Endoscopy* , reporting on interesting cases and new techniques in gastroenterological endoscopy. All papers include a high-quality video and are published with a Creative Commons CC-BY license. Endoscopy E-Videos qualify for HINARI discounts and waivers and eligibility is automatically checked during the submission process. We grant 100% waivers to articles whose corresponding authors are based in Group A countries and 50% waivers to those who are based in Group B countries as classified by Research4Life (see: https://www.research4life.org/access/eligibility/ ). This section has its own submission website at https://mc.manuscriptcentral.com/e-videos
